# Proteins Involved in the Trafficking and Functional Synaptic Expression of AMPA and KA Receptors

**DOI:** 10.1100/tsw.2002.97

**Published:** 2002-02-22

**Authors:** Sarah A. De La Rue, Jeremy M. Henley

**Affiliations:** MRC Centre for Synaptic Plasticity, Department of Anatomy, School of Medical Sciences, University Walk, University of Bristol, Bristol, BS8 1TD, UK

**Keywords:** AMPA receptors, glutamate receptors, kainite receptors, PICK1, NSF, Narp, Mint1, MAGUKs, PDZ domain, GRIP, ABP, SAP97, PSD-95, Stargazin, 4.1N

## Abstract

α-Amino-3-hydroxy-5-methylisoxazolepropionate receptors (AMPARs) mediate the majority of fast synaptic transmission in the mammalian central nervous system, play a central role in synapse stabilisation and plasticity, and their prolonged activation is potently neurotoxic. The functional roles of kainate receptors (KARs) are less well defined but they play a role in some forms of synaptic plasticity. Both receptor types have been shown to be highly developmentally and activity-dependently regulated and their functional synaptic expression is under tight cellular regulation. The molecular and cellular mechanisms that regulate the synaptic localisation and functional expression of AMPARs and KARs are objects of concerted research. There has been significant progress towards elucidating some of the processes involved with the discovery of an array of proteins that selectively interact with individual AMPAR and KAR subunits. These proteins have been implicated in, among other things, the regulation of post-translational modification, targeting and trafficking, surface expression, and anchoring. The aim of this review is to present an overview of the major interacting proteins and suggest how they may fit into the hierarchical series of events controlling the trafficking of AMPARs and KARs.

## INTRODUCTION

Glutamate is the major excitatory neurotransmitter throughout the mammalian brain[1]; glutamate receptors have been suggested to play a central role in learning and memory[2]. Furthermore, dysfunction of glutamatergic neurotransmission has been implicated in a wide variety of disease states and in neurotoxicity associated with damage from neurological insults and degenerative diseases.

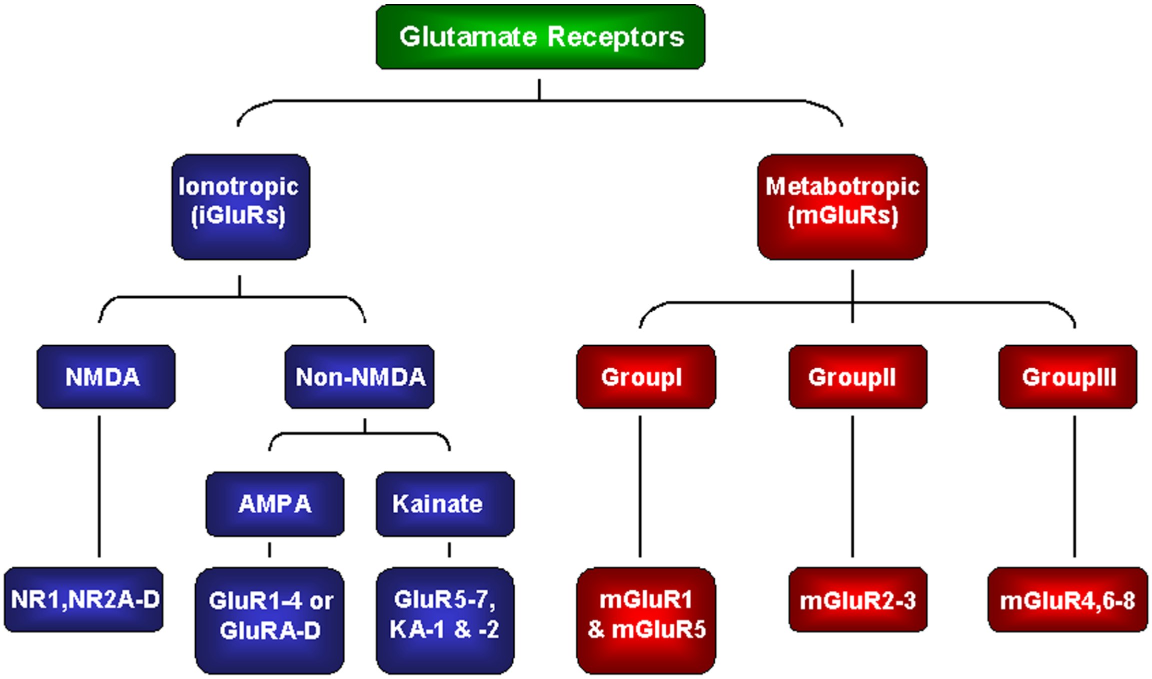

**FIGURE 1.** Schematic illustrating the relationships between members of the glutamate receptor family.

Glutamate receptors fall into two major categories: ligand-gated or ionotropic and guanine-nucleotide-binding protein (G-protein) coupled or metabotropic (Fig. 1). Ionotropic receptors become permeable to cations upon binding of ligand, the metabotropic receptors transduce a signal through their associated G-proteins upon binding of ligand. This review focuses on two subtypes of ionotropic glutamate receptors: α-amino-3-hydroxy-5-methyl-4-isoxazolepropionic acid receptors (AMPARs) and kainate receptors (KARs). There is now considerable information available about the other subtype of ionotropic receptor, the N-methyl-D-aspartate receptor (NMDAR), adequate discussion of which warrants a separate review.

Glutamatergic synapses, especially those in the hippocampus and cerebellum, have been extensively studied. The mature synapse consists of a presynaptic terminus directly apposed to the postsynaptic membrane, which is characterised by an electron dense thickening known as the postsynaptic density (PSD). The PSD comprises receptors and the associated intracellular proteins involved in their scaffolding, targeting, and regulation, as well as a wide range of signalling proteins that activate intracellular signal cascades. In many areas of the brain, the majority of glutamatergic postsynapses are located on dendritic spines (for review see Sorra and Harris[3]). The spine serves to increase the surface area and therefore the number of connections that an individual dendrite can make. In addition, spines have functional implications, serving both to localise the spread of calcium ($Ca^{2+}$) and the flow of potential from the synapse to the dendrite and therefore neighbouring synapses.

Theoretically, many thousands of receptor variants are possible arising from different combinations of subunits, each of which can be spliced and/or edited and/or modified by post-translational processes. There must therefore be a high order of complexity in the manufacture and localisation of AMPARs and KARs. How the appropriate subunit combination is assembled and targeted to the correct cellular location at the right time is a major outstanding question in Neuroscience. Increasing attention is now focusing on how these processes occur. A useful analogy is a postal system in which particular AMPAR or KAR complexes are delivered to the correct address at the appropriate time. How is this achieved? Do particular receptor complexes contain the specific identifying tags, and do specific synapses, or types of synapses (for example those that are newly formed, or highly active, or that synapse with particular types of other neurones) possess selective identifiers analogous to postcodes or Zip codes?

This is a multilayered problem that appears to involve complex spatio-temporal coordination of multiple protein interactions. While some progress has been achieved in identifying many of the proteins that bind directly to AMPAR and KAR subunits and the functional consequences of blocking some of these interactions have been determined, the overall picture remains unclear. For example, in most cases so far reported, blockade of specific AMPAR-interacting proteins appears to prevent only the last stages of receptor insertion into the postsynaptic membrane. The AMPARs arrive at the dendritic spine, but they fail to be surface expressed at the synapse. What then are the proteins involved in transporting, address selection, internalisation, and recycling or degradation? In a recent paper it has been suggested that one protein, synapse-associated protein of 97kD (SAP97), interacts with AMPARs early in the secretory pathway, while the receptors are in the endoplasmic reticulum or cis-Golgi[4]. The aim of this article is to present an overview of the major interacting proteins described so far and to place these in the context of how they may participate in the well-ordered series of events controlling the cell biology of AMPARs.

## MOLECULAR PROPERTIES OF AMPARS AND KARS

### AMPARs

#### *Importance*

AMPARs mediate the overwhelming majority of fast excitatory neurotransmission in the mammalian central nervous system. Their involvement in the mechanisms underlying synaptic stabilisation and plasticity is now well established[1,2,5]. Overactivation of AMPARs is potently neurotoxic; their dysfunction has been implicated in many clinically important disease states including epilepsy and neurodegenerative diseases such as Alzheimer’s and Parkinson’s[6,7,8].

#### *Subunits and Topology*

AMPARs are multimeric assemblies of the subunits GluR1-4[9]. Each subunit comprises ~900 amino acids and has a molecular weight of ~105 kDa. The N-terminus is extracellular; there are three membrane-spanning and one re-entrant loop domains and the C-terminus is intracellular[10,11]. In addition, 50 to 70% of AMPARs are intracellular[12,13], with a significant proportion being localised within dendrites[14,15].

The topology of the AMPAR subunits is shown in schematic form in Fig. 2[9,11]. Each subunit is composed of five hydrophobic domains, the first of which is at the N-terminus and forms a signal peptide for appropriate membrane insertion. This domain is cleaved from the mature protein. The remaining four hydrophobic regions incorporate into the lipid membrane. Importantly, three span the membrane whereas the second hydrophobic region constitutes a re-entrant loop that enters the lipid bilayer from the intracellular face, then forms a loop structure and exits from the same side[16]. This membrane loop lines the pore in the mature channel. The N-terminal end of AMPAR subunits forms a large extracellular domain that is involved in hetero-oligomerisation of the subunits to form the receptor channel[17,18].

Juxta-membrane regions and the C-terminal part of the extracellular loop have also been implicated in assembly of receptor complexes[18]. The channels have been reported to consist of a combination of either four or five subunits, or possibly both[18,19,20,21,22]. The ligand-binding site of each subunit is formed in combination between the extracellular N-terminal domain and the extracellular loop[23]. The N-terminus has also been shown to be involved in AMPAR clustering through the extracellular interacting protein neuronal activity-regulated pentraxin (NARP[24]; see below). The extracellular loop comprises approximately 173 amino

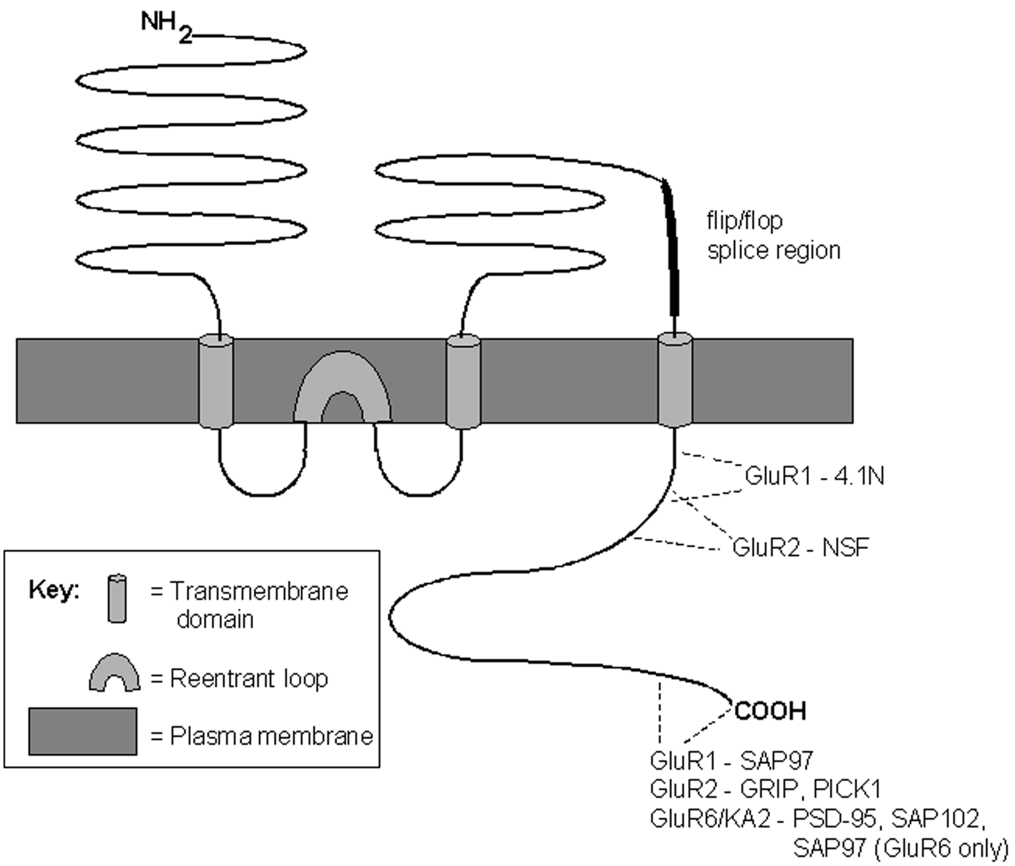

**FIGURE 2.** Schematic showing the conserved membrane topology of AMPAR and KAR subunits. The sites of protein–protein interactions have been indicated where known.

acids, and has been shown to control various aspects of receptor function. The intracellular C-terminal domain has been most extensively studied in terms of identifying protein interactors that may be involved in trafficking, targeting, and anchoring of AMPARs. An in-depth discussion is given in later sections of this review.

## Editing

For the GluR2 subunit, the re-entrant loop is the site of RNA editing, an important post-transcriptional event that has profound effects on receptor function[25]. This is the so-called Q/R switch where a single residue in position 586 of the loop is edited from C<u>A</u>G (glutamine/Q) to C<u>G</u>G (arginine/R). This Q/R editing event appears to occur only in the GluR2 AMPAR subunit[26]. The edited version of GluR2 displays markedly different rectification properties, single channel conductance, and $Ca^{2+}$ permeability. Homomeric GluR1, 3, and 4 channels all show inward rectification in their cation currents, as do unedited GluR2 channels[27]. However, edited homomeric GluR2 channels, or heteromeric channels that contain the edited GluR2 subunit, show linear or slightly outwardly rectified currents and decreased single channel conductance at negative membrane potentials. Also, AMPARs that do not contain an edited GluR2 subunit are $Ca^{2+}$ permeable whereas those that do are relatively impermeable to $Ca^{2+}$. The GluR2 subunit is dominant in heteromeric channels. Although low levels of unedited GluR2 are found in the foetal brain, all GluR2 appears to be edited in the adult brain[28].

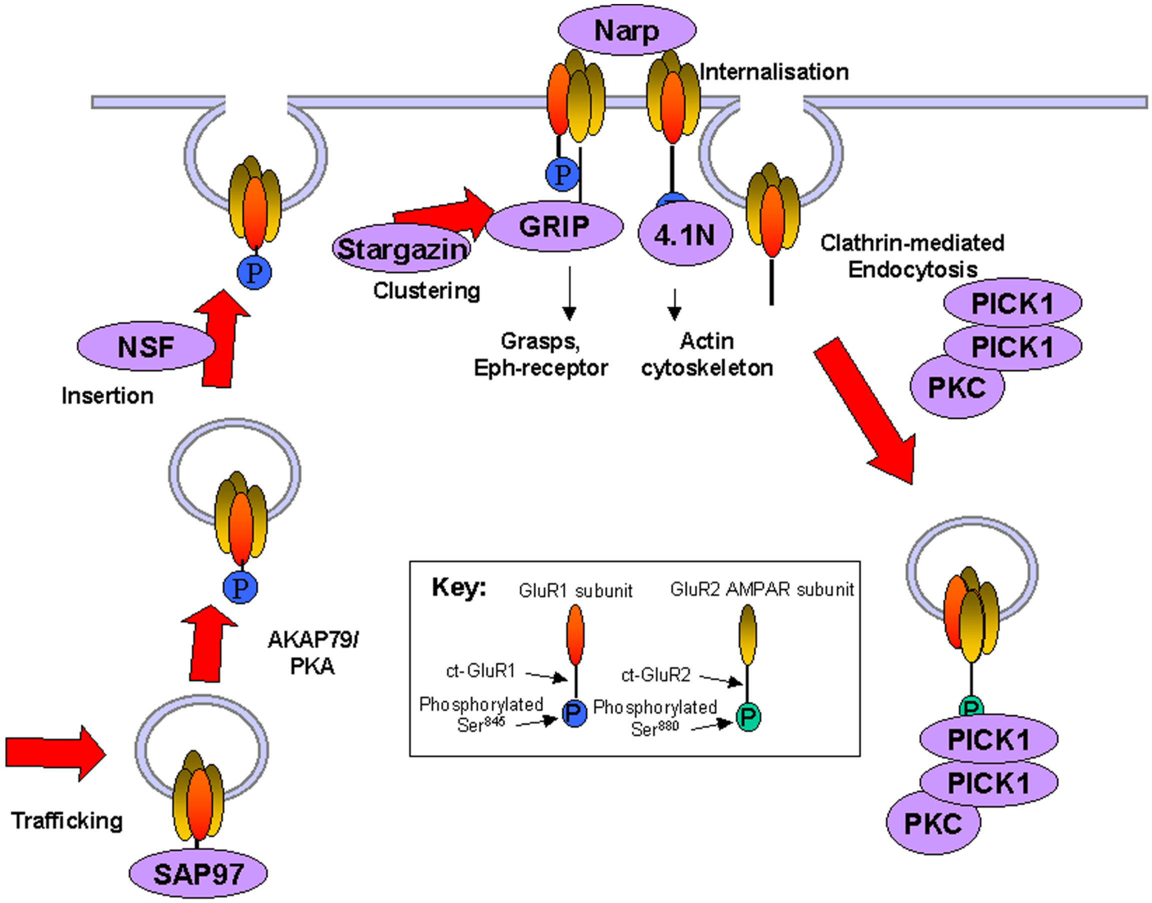

**FIGURE 3.** Simplified model showing the possible roles of individual AMPAR interacting proteins.

### *Splicing*

In addition to forming part of the ligand-binding site, the extracellular loop also contains a cassette for which two distinct splice variants exist, flip and flop[29]. Although these variants show no differences in pharmacological selectivity, homomeric flip channels are activated considerably more efficiently by glutamate than flop channels. In addition, they show different desensitisation kinetics. For all subunits, the flip splice variant is expressed prominently before birth and continues to be expressed at similar levels throughout development and into adulthood, whereas expression of the flop splice variant increases throughout development and reaches adult levels at postnatal day 14[30].

## KARs

### *Importance*

While the physiological roles of AMPARs and NMDARs in synaptic transmission and synaptic plasticity are relatively well established (see [2]), despite some recent progress, the physiological roles of KARs in the vertebrate central nervous system are largely unknown (for review see [31,32,33]).

### Subunits and Topology

Low-stringency, homology-based screening with the other glutamate receptor subunits was used to clone the five identified KAR subtypes[34]. The KAR subunits are ~40% homologous to AMPAR subunits and ~20% homologous to NMDAR subunits. The homology within the GluR5–7 subclass is 75 to 80%, whereas it is 68% between KA1 and KA2. However, the homology between the two subclasses is 45%, barely greater than with the AMPAR. The GluR5–7 subunits can each form functional homomeric, kainate-gated receptors when expressed in heterologous cell lines[35,36]. KA1 and KA2, on the other hand, cannot form functional homomeric channels but can form part of functional heteromeric channels with members of the other subclass[37,38]. However, KA1 and KA2 have a significantly higher affinity for kainate than GluR5–7. These are therefore likely to correspond respectively to the high- and low-affinity kainate binding sites identified in early radiographic studies[39,40]. Their topology has not been directly investigated, but is likely to be the same as the AMPAR subunits as the major structural features of the hydrophobic regions and the ligand-binding domain are conserved.

### Editing

Like the AMPAR GluR2 subunit, GluR5 and 6 undergo mRNA editing at the tip of the pore-lining membrane loop at the Q/R site[35]. This affects rectification properties and $Ca^{2+}$-permeability, with the additional effect of increasing chloride permeability. In addition, the GluR6 subunit undergoes splicing at two additional sites in the TM1 region[41]. This has the effect of completely abolishing passage of $Ca^{2+}$. This splicing is developmentally regulated, with a significant proportion of edited receptors present both in embryonic and adult brains[34,42]. However, unlike for GluR2, this editing does not go to completion with several edited variants existing across different sites within the same neurone[43].

### Splicing

Both the GluR5 and GluR7 subunits undergo alternative splicing at their C-terminus[44,45], which, in an analogous fashion to the AMPAR subunits, is likely to have implications for interacting proteins and therefore differential trafficking. The other subunits have not been shown to have splice variants in the rat, although two splice variants have been found for the mouse GluR6 subunit, again at the C-terminus[46].

## AMPAR and KAR Trafficking and Surface Expression

Developmental and activity-dependent changes in the functional synaptic expression of AMPARs are under tight cellular regulation, and the molecular mechanisms that control the postsynaptic insertion (exocytosis), arrangement, and internalisation (endocytosis) of AMPARs are the subject of widespread investigation (for reviews see Morales and Goda[47], Malinow et al.[48], Sheng and Lee[49], and Carrol et al.[50]). Biochemical assays using $^{35}$S-methionine labelling and surface biotinylation determined the metabolic half-life and the half-life of surface expression of AMPARs both to be in the order of 30 h[51,52,53]. Electrophysiological evidence, however, has suggested that the AMPAR surface expression is highly dynamic (for review see Morales and Goda[47]). For example, at developmentally early stages it has been demonstrated that some synapses contain functional NMDARs but do not exhibit AMPAR-mediated responses. These are termed *silent synapses* since at physiological resting potentials the NMDA receptors are blocked by magnesium and thus do not pass currents when exposed to glutamate. This results in synapses that are functionally silent under conditions of normal synaptic transmission. Stimuli that induce NMDA receptor-dependent long-term potentiation(LTP; [2]) rapidly lead to the appearance of functional AMPARs at these synapses[54,55,56].

A percentage (50–70) of AMPARs are intracellulal[12,57,58] and a significant proportion of intracellular AMPARs are in dendrites[14,15]. In hippocampal slice cultures it has been reported that 90% of AMPARs are localised to the dendritic shaft, but only 20% of these are at the cell surface[59]. Furthermore, approximately 10% of AMPARs are localised in the dendritic spine, with 7% of these being intracellular, leaving only 3% expressed at the surface of the dendritic spine. These numbers indicate that a large amount of AMPARs are held extrasynaptically but in a position where they can be rapidly recruited into the synapse, either extrasynaptically at the surface of the cell or intracellularly within the spine. Therefore, a simple model to explain this observation is that AMPARs are recruited to the postsynaptic membrane from intradendritic stores. The role of fusion at the postsynaptic membrane in the establishment of LTP has been revealed by using inhibitors of several parts of the exocytotic machinery to reduce LTP and by enhancing LTP via infusion with a recombinant constituent of the vesicle cycling machinery[60].

Further support for the hypothesis that AMPARs are highly dynamic has come from recent work indicating that an interaction between the GluR2 subunit and N-ethylmaleimide sensitive factor (NSF) plays an important role in maintaining the pool of functional AMPARs[61], and that blockade of the interaction leads to a rapid run-down (50% in ~10 min) in AMPA-mediated synaptic excitatory postsynaptic currents. Expression of a peptide that specifically prevents the binding of NSF to the AMPAR, but not of a control peptide, in cultured hippocampal neurones resulted in a dramatic loss in the number of AMPAR aggregates on the cell surface[62]. Importantly, AMPAR aggregates were still present close to synapses inside the dendrites and the total amount of GluR2 immunoreactivity did not decrease[62]. These findings suggest that blocking the interaction between NSF and GluR2 does not prevent receptor synthesis or passage through the endoplasmic reticulum/Golgi systems but rather that it has an effect at the synapse.

In addition to electrophysiological studies, much recent work on the trafficking and surface expression of AMPARs has relied on the use of specific antibodies directed against extracellular and intracellular domains of AMPAR subunits. These types of studies have provided independent evidence for the existence of silent synapses[63,64] and have also yielded corroborative evidence to suggest that certain forms of LTP involve the insertion of intracellular AMPARs into the plasma membrane at these silent synapses[65,66].

Another set of studies[67,68] used FM1-43 (a fluorescent dye with a high affinity for lipids, which can be visualised using light microscopy) to label vesicles within the cellular processes of cultured hippocampal neurones. They found that a prolonged exposure of the cultured neurones to FM1-43–labelled vesicles that undergo calcium-evoked dendritic exocytosis. Vesicles labelled by this prolonged exposure were found to colocalise with a trans-Golgi network marker, implying an origin associated with biosynthetic pathways, and some were found to lie at the base of putative spine precursors. Additionally, they established a direct link between $Ca^{2+}$/calmodulin-dependant protein kinase II (CaMKII) activity and dendritic exocytosis. These observations are consistent with previous reports demonstrating that CaMKII is both necessary and sufficient for LTP to occur[69,70]. These studies link vesicle exocytosis with the establishment of LTP.

Other recent advances towards better understanding the molecular mechanisms of synaptic plasticity have come from viral transfection of neurones with green fluorescent protein (GFP)–tagged GluRs. Tetanic synaptic stimulation induced a rapid NMDAR-dependent delivery of GFP-GluR1 into dendritic spines[71]. It has been shown that native AMPARs in hippocampal neurones are assembled from combinations of mainly GluR1/GluR2 or of GluR2/GluR3[72,73]. Based on data obtained using GFP-tagged subunits, it has been proposed that there are differential targeting mechanisms for AMPARs comprising either GluR1/GluR2 or GluR2/GluR3 subunit assemblies. GluR1/GluR2 receptors are added to synapses during plasticity, a process that requires interactions between GluR1 and group I PDZ domain proteins. In contrast, GluR2/GluR3 receptors replace existing synaptic receptors in a constitutive manner dependent on interactions by GluR2 with NSF and group II PDZ domain proteins[74]. While these studies have provided valuable information, it is difficult to distinguish between surface-expressed and intradendritic localisation of the GFP fluorescence. Recently, another study using a thrombin cleavage assay to measure the rate and location of surface expression of homomeric AMPAR reported broadly consistent results[75]. This study showed that surface insertion of the GluR1 subunit under basal conditions was relatively slow but was stimulated by NMDAR activation. In contrast, GluR2 exocytosis was rapid and constitutive. GluR1 was inserted initially at extrasynaptic sites; GluR2 was inserted more directly at synapses. These data have been used to propose that the combination of regulated addition and continuous replacement of synaptic receptors may be the mechanism for stabilising long-term changes in synaptic efficacy and could be a general model for how the surface AMPAR number is established and maintained.

Recent studies have focused on identifying the molecular basis of AMPAR recycling. The internalisation of AMPAR from the surface of the synapse is mediated by the formation of clathrin-coated pits[76]. Disruption of clathrin-mediated endocytosis — for example, by biochemical inhibition of pit formation or overexpression of a dominant negative form of dynamin — effectively blocks the internalisation of AMPAR[77,78]. This model is further supported by the observation that botulinum toxin (which blocks exocytosis) causes a reduction in AMPAR responses[79].

AMPAR turnover appears to be directly regulated by synaptic activity, with removal from the synaptic membrane occurring in a ligand-dependent manner. Increasing synaptic activity using picrotoxin, a γ-aminobutyric acid receptor antagonist, has been shown to cause a decrease in the number of surface-expressed AMPAR and the size of AMPAR clusters in cultured neurones[80]. Conversely, decreasing synaptic activity using tetrodotoxin (sodium [$Na^+$]-channel blocker) or AMPAR antagonists causes an increase in synaptic AMPAR[78]. Exogenous application of glutamate or AMPA causes internalisation and redistribution of AMPARs, but probably in a subset of synapses only, as miniature excitatory postsynaptic currents decrease in frequency but not size[80,81,82]. This can occur in both an NMDAR-dependent and -independent fashion, with differential implications for the fate of internalised receptors. AMPARs internalised in response to NMDAR activation are rapidly recycled and reinserted into the synaptic membrane; in contrast, treatment with AMPA causes the internalised receptors to be targeted to protein degradation pathways[78,83].

The mechanisms by which this dynamic regulated surface expression is achieved are still unclear. Some of the many proteins that interact with the C-terminal regions of AMPAR could function to stabilise receptors at the surface of the synapse, with activity differentially regulating their ability to bind or their affinity for the receptor. Alternatively, activity could trigger endo- or exocytosis or cause a change in the constitutive rate. The indication is that all of these strategies are involved at least to some degree. It has been shown that the exocytosis of putative AMPAR-containing vesicles occurs in response to NMDAR activation[67,68]. More recently, differential binding of proteins to the C-terminus of GluR2 depending on phosphorylation state has been demonstrated[84] in a $Ca^{2+}$ and therefore activity-dependent fashion.

Taken as a whole, these data suggest a model that involves constitutive turnover of AMPAR from a pool close to the synapse. LTP or its functional inverse, long-term depression, could then be mediated by a change in the size of this recycling pool, by the involvement of a separate pool of receptors or a change in the rate of either the insertion or removal of receptors from the synaptic membrane. This could occur by means of the mobilisation of a cytoplasmic pool of receptors or by the diffusion of extrasynaptic surface AMPAR into the synaptic cleft. There is currently little evidence to indicate a preference for either model. One consideration, however, is the presence of the PSD, which appears to be a dense raft of closely interacting proteins that intuitively would seem to form a barrier to the rapid insertion of receptor transport vesicles into the synaptic plasma membrane (but see Ziff[85]).

How is this achieved? The answer lies in the complex series of protein interactions alluded to above. In this review we describe the proteins that interact with these receptors and, where information is available, outline what role(s) they may play in the regulation of AMPAR and KAR trafficking, surface expression, anchoring, and internalisation.

## INTRACELLULAR C-TERMINAL DOMAINS OF AMPARS AND KARS AS SITES FOR PROTEIN–PROTEIN INTERACTION

As discussed earlier, within neurones, AMPARs are clustered at postsynaptic sites[72,86] but a significant proportion are also present in the cytoplasm including within the dendritic cytosol[12,57,58,86,87,88,89]. Since at least part of this intracellular pool of receptors is localised in the dendrites and spine apparatus[14,15] it is probable that changes in the surface expression of AMPARs can come from a rapid translocation of receptors to and from an intracellular pool that is closely associated with the postsynaptic membrane. Indeed, recent evidence has suggested that the surface expression of AMPAR is a highly dynamic system: for example, in the conversion of silent synapses that lack AMPARs into mature, functional synapses[54,55,56]. A simple model to explain this observation is that AMPARs are recruited to the postsynaptic membrane from closely apposed intradendritic stores.

Because the C-terminal domains of AMPAR and KAR subunits are the predominant cytosolic regions, they are likely to be a major factor in the trafficking of the AMPARs. As such, they have been the subject of intensive investigation in the search for interacting proteins, mainly using yeast two-hybrid (Y2H) and glutathione-S-transferase pull-down assays. Several protein interactors have been isolated and, in some cases, the roles of individual interactors in receptor trafficking and surface expression have begun to be elucidated. It is becoming increasingly clear that AMPAR complexes get "handed" from one interacting protein to another in a sequential and probably hierarchical process and these interacting proteins can act as the sorting and delivery mechanisms for the correct delivery to and anchoring at appropriate synapses.

## PROTEINS KNOWN TO INTERACT WITH AMPARS AND KARS

### PDZ Domain–Containing Proteins

A protein–protein interaction domain that has been implicated in the anchoring of many different receptors is the PDZ domain, which is named after the first three proteins identified to contain it: postsynaptic density protein-95 (PSD-95), Discs-large, and Zona occludens-1[90]. PDZ domain–containing proteins interact with all the major classes of glutamate receptors. PDZ domains are approximately 90 amino acid domains that form a rigid three-dimensional structure[91,92] incorporating a specialised, selective-binding pocket.

Most PDZ-mediated interactions occur via the recognition of a short motif of three to seven residues located at the extreme C-terminus of the binding protein[93,94]. PDZ-binding domains mainly fall into two classes: $E^{S}/_{T}X^{V}/_{I}$ (type I) and $\phi X \phi$ (type II), where *X* is any amino acid and $\phi$ a hydrophobic residue. Some proteins, however, interact with both type I and type II PDZ-binding domains. For example, protein interacting with C-Kinase (PICK1; see below) binds protein kinase C (PKC)$\alpha$ at a type I site [95] but also mGluR7a and AMPAR subunits at type II sites[96,97,98,99,100].

A family of PDZ domain–containing proteins called the *membrane-associated guanylyl kinase proteins* (MAGUKs) has been the subject of intensive study both for its role in anchoring and the recruitment of signal transduction molecules to the receptor complex[101]. Members of this family each contain three PDZ domains, as well as an Src homology 3 (SH3) and a guanylyl kinase protein–protein interaction domain. The Discs-large protein has been shown to be directly responsible for the targeting of proteins such as the Shaker voltage-gated potassium channel to the *Drosophila* neuromuscular junction[102]. The PSD-95 family of MAGUKs has a less clearly defined targeting role in synaptic localisation of the NMDAR, but has been shown to play a role in modulating the electrophysiological properties of the receptor as well as recruiting second-messenger responsive proteins that initiate downstream signalling pathways[103].

Many other proteins contain variable numbers of PDZ domains as well as many other protein–protein interaction domains, and these perform a whole variety of functions with respect to their target proteins.

## AMPARs

### *Glutamate Receptor Interacting Proteins 1 and 2 (GRIP1 and -2) and AMPAR Binding Protein (ABP)*

GRIP was the first AMPAR-interacting protein to be identified using the Y2H screen[104] and was subsequently shown to interact with the short isoform of GluR2 and with GluR3. It is a 130-kDa protein that contains seven PDZ domains, of which domains 4 and 5 mediate binding to the extreme C-terminal ESVKI motif of GluR2 and -3. Deletion of the last seven amino acids of GluR2 prevents this interaction, and the last ten amino acids of GluR2 are all that is required[104].

GRIP has been shown to bind to other receptor proteins; for example, the Eph-receptor family and their membrane-bound ligands, the ephrins, both bind to GRIP[105,106]. Eph-receptors are receptor tyrosine kinases that bind to the sixth and seventh PDZ domains of GRIP. Interactions between Eph-receptors and their ephrin ligands on adjacent cells are important for processes involved in neurite extension and axonal guidance[107].

The function of GRIP is not fully resolved, although multiple PDZ domains suggest that it serves a scaffolding function similar to that of PSD-95[104]. For example, the PDZ domains other than 4 and 5 could provide a mechanism to link AMPARs to other binding partners of GRIP. Indeed, several GRIP-associated proteins (GRASPs) that bind to distinct PDZ domains within GRIP have been identified[108]. Of these, GRASP-1 is a neuronal rasGEF associated with GRIP and AMPARs *in vivo.* Overexpression of GRASP-1 in cultured neurones specifically reduced the synaptic targeting of AMPARs. The subcellular distribution of both AMPARs and GRASP-1 is regulated by NMDAR activation.

ABP was isolated in similar fashion to GRIP[109] and found to share 64 to 93% homology with GRIP within the six PDZ domains that it shares. GRIP2 is probably a longer splice variant of ABP, with complete sequence identity over the six PDZ domains but containing an additional seventh PDZ domain[110]. ABP and GRIP1/2 colocalise with GluR2 at synapses[109,111], although they are found additionally within the dendrites and soma of cultured neurones[104,112]. ABP has been shown to be distributed widely throughout the central nervous system but also to be enriched in the PSD[109].

The fourth to sixth PDZ domains of ABP are involved in multimerisation of GRIP with ABP; the other PDZ domains are available to recruit other as-yet-unidentified proteins. An extensive raft of proteins could assemble in this fashion. Alternatively, if they were involved in trafficking to the synapse, other chaperoning factors could bind there. A peptide that selectively blocks GluR2 binding to ABP and to GRIP1/2 accelerates GluR2 endocytosis at the synapse[113], supporting the hypothesis that ABP and GRIP1/2 play a role in anchoring or attenuating AMPAR at the synaptic membrane.

### *PICK1*

PICK1 was originally identified by Y2H screening as a protein that interacts with the catalytic subunit of PKCα through its single PDZ domain[95]. It is ubiquitously expressed, but is expressed at higher levels in the brain and testis[95]. It has subsequently been shown to interact with GluR2 and GluR3[98,114] via the same extreme C-terminal PDZ-binding domain as ABP/GRIP1/2. However, the requirements for recognition within this ten-amino-acid stretch are different for PICK1 and GRIP. A single amino-acid substitution in the PDZ binding motif, in which serine 880 is replaced with a glutamate, stops recognition by GRIP whereas PICK1 can still bind.

Domain analysis of the PICK1 protein has identified a row of nine acidic residues at the C-terminus (amino acids 382 to 390) and three possible PKC phosphorylation sites, one of which occurs within a P-loop ATP/GTP binding motif (GxxxxGKS/T) normally found in the RAS protein family and in guanylate-, thymidine-, and thymidylate kinases. There is a putative carboxylate-binding loop within the PDZ domain near the N-terminus of the protein; it is this carboxylate binding which is believed to interact with the t/sXV motif on PDZ-binding proteins[115]. A double mutation of the PICK1 carboxylate-binding loop (residues lys27 and asp28 to alanines) inhibits PICK1 interaction with the PKCα.

PICK1 has also been shown to be capable of dimerisation through a different site, which allows it to aggregate target proteins. This has already been demonstrated for AMPARs in heterologous-expression systems[96,98]. PICK1 has been shown to complex with PKCα upon tissue plasminogen activator (activates PKCα) induction; these complexes are targeted to PICK1-GluR2 complexes in spines. It has been demonstrated that GluR2 can be phosphorylated on serine 880 by PKCα[116] (some implications are discussed later in this paper). This targeting of PICK1-PKCα complexes to GluR2 in spines causes a decrease in surface GluR2 consistent with a PICK1/PKCα-mediated release of GluR2 from GRIP/ABP binding.

As discussed above, modulation of the surface expression of AMPARs is a key process underlying NMDAR-dependent synaptic plasticity. NMDA application to cultured hippocampal neurones markedly reduces the amount of [$^3$H]AMPA bound to surface-expressed receptors of the hippocampal neurones but not to their total membrane fraction[117]. This process was mimicked by a PKC activator, phorbol ester, but blocked by an inhibitor of the same kinase, calphostin C. The NMDA treatment also markedly altered the interaction between the AMPAR subunits and PICK1 with a PKC-dependent enhancement of the association of GluR2/3 with PICK1. Viral expression of GFP-tagged C-terminal domain of GluR2 (GFP-ct-GluR2) specifically and significantly blocked the NMDA-triggered reduction in [$^3$H]AMPA binding, whereas expression of GFP-ct-GluR1 had no effect. Coimmunoprecipitation using anti-PICK1 antibodies confirmed that GFP-ct-GluR2 prevented the PICK1-GluR2/3 interaction consistent with the hypothesis that NMDA-induced down regulation of functional AMPARs involves the interaction between GluR2/3 subunits and PICK1[117]. Furthermore it has been shown that PICK1 also binds in neurones and in heterologous cells to PKCα and that the interaction is highly dependent on kinase activation[116]. The formation of PICK1-PKCα complexes is strongly induced by tissue plasminogen activator, and PICK1-PKCα complexes are cotargeted with PICK1-GluR2 complexes to dendritic spines, where GluR2 is phosphorylated by PKC on serine 880. These workers propose that PICK1 functions as a targeting and transport protein that directs the activated form of PKCα to GluR2 in spines, leading to the activity-dependent release of GluR2 from synaptic anchor proteins and the PICK1-dependent transport of GluR2 from the synaptic membrane.

### *SAP97*

SAP97 is a member of the synapse associated protein (SAP) family. Other members include SAP90 (PSD-95), chapsyn110 (PSD93), and SAP102, which interact with NMDAR subunits. SAP97 has a presynaptic localisation[118] where its function remains unclear. In addition, however, SAP97 can interact selectively with postsynaptically localised GluR1 in coimmunoprecipitation and cross-linking experiments from the rat brain[119]. Indeed, SAP97 is the only PDZ protein known to interact with GluR1. SAP97 concentrates at synapses that contain

GluR1, but not necessarily GluR2 or GluR3. It is most highly concentrated within the PSD, suggesting that SAP97 may help to anchor GluR1-containing AMPARs at the synapse[120]. It is expressed in the hippocampus and along bundles of unmyelinated axons throughout the brain[118]. Another study has indicated that phosphorylation of AMPARs is enhanced by a SAP97-AKAP79 complex that directs PKA to GluR1 via a PDZ-domain interaction[121]. SAP97 has also been suggested to play a role in the provision of new anchoring sites at synapses via a mechanism that involves the binding of autophosphorylated CaMKII to NMDARs. In turn, SAP97 binds to CaMKII; it also provides the scaffolding for retaining new GluR1-containing AMPARs[122].

However, a recent report has indicated that interactions involving SAP97 and GluR1 occur early in the secretory pathway, while the receptors are in the endoplasmic reticulum or cis-Golgi; see Sans et al.[4]. Few synaptic GluR1-containing receptors were found to associate with SAP97, suggesting that SAP97 dissociates from the receptor complex at the plasma membrane. These workers also demonstrate that NMDAR-evoked internalisation of GluR1 does not require SAP97, which suggests that GluR1-SAP97 interactions are involved in AMPAR targeting but not in exo- or endocytosis.

### *Mint1 (LIN-10, X11)*

The Mint family consists of evolutionarily conserved PDZ-containing adapter proteins from *Caenorhabditis elegans* to mammalian neurones. Mint1 and LIN-10, a homologue in *C. elegans,* comprise macromolecular complexes in the pre- and postsynaptic terminals, thereby bringing synaptic vesicles to the exocytotic transmitter release site and localising receptors and ion channels in the specific membrane domains[123]. Vesicles containing NMDAR-2B (NR2B subunit) are transported along microtubules by KIF17, a neurone-specific molecular motor in neuronal dendrites. That selective transport is accomplished by direct interaction of the KIF17 tail with a PDZ domain of Mint1 that is a constituent of a large protein complex including CASK and the NR2B subunit[124].

In *C. elegans* GLR-1 are AMPA-type glutamate receptors that are expressed postsynaptically at target cells of the sensory neurone ASH and are required for ASH-mediated touch sensitivity[125,126]. The localisation of these receptors has been shown to be dependent on the interaction with LIN-10[127], the *C. elegans* orthologue of Mint1/X-11[128,129]. It has been proposed that the protein X-11 may be involved in the localisation of GluRs at mammalian synapses. However, although the mammalian protein X-11 can restore the correct localisation of GLR-1 in mutant *C. elegans* lacking LIN-10[127], the involvement of X-11 in the synaptic localisation of mammalian GluRs has not been demonstrated.

## KARs

KARs are of particular interest because, unlike AMPARs — which appear to be predominantly or exclusively postsynaptic — functional KARs have both pre- and postsynaptic localisations (for reviews, see Chittajallu et al.[33] and Lerma et al.[34]). There have been fewer reports of proteins that interact with KARs, and the mechanisms of KAR targeting and clustering are less well defined. In terms of regulation, it has been shown that GluR6 expressed in heterologous cells can be phosphorylated by cyclic adenosine monophosphate–dependent protein kinase (PKA). Kainate-evoked currents are potentiated by intracellular perfusion of PKA due to phosphorylation of GluR6 at the serine residue, Ser 684[130,131]. However some PDZ-binding proteins have been identified.

### *PSD-95 (SAP90) and SAP102*

Using coimmunoprecipitation assays, PSD-95 and SAP102 were isolated as interacting proteins for KA2 and GluR6 (see Garcia et al.[132]). However, SAP102 coimmunoprecipitates with GluR6 (but not KA2). GluR6 clustering is mediated by the interaction of its C-terminal amino acid sequence, ETMA, with the PDZ1 domain of PSD-95. In contrast, the KA2 C-terminal region binds to, and is clustered by, the SH3 and guanylyl kinase domains of PSD-95. Coexpression of GluR6 or GluR6/KA2 KARs with PSD-95 alters receptor function by reducing desensitisation.

In a subsequent study, these workers showed that *in vivo* PSD-95 binds tightly to KAR subunits, while SAP102 is only weakly associated, suggesting that this glutamate receptor differentially associates with PSD-95 family members. They suggest that differential association of KARs with SAP family proteins may be one mechanism of directing the differential subcellular localisation of KARs containing different subunits[133].

## Other PDZ Interactors with KARs

To carry out a systematic search for KAR-interacting proteins we have performed Y2H and glutathione-S-transferase pull-down assays with the C-terminal domains of splice variants $GluR5_{2a}$, $GluR5_{2b}$, and $GluR5_{2c}$, as well as with the C-terminal domain of GluR6 (results are not published). No interactors were detected with $GluR5_{2a}$, which does not contain a PDZ-binding motif. $GluR5_{2b}$, $GluR5_{2c}$, and GluR6 do contain consensus motifs for PDZ protein interactions and were found to interact with four separate PDZ-containing proteins, namely syntenin, PICK1, GRIP, and PSD-95. Biochemical and immunocytochemical experiments verified the interactions. We show that blockade of GRIP binding causes a decrease in KAR-mediated excitatory postsynaptic currents (Hirbec et al., manuscript in preparation).

## Non-PDZ Domain–Containing Proteins

### *AMPARs*

#### *NSF*

The N-methylmaleimide sensitive factor (NSF) ATPase was originally characterised through its involvement in protein transport activity of the Golgi membranes. Subsequently it was shown to be involved in the endoplasmic reticulum to Golgi transport and endosome fusion[134]. Later a central role for NSF was identified in the docking and/or fusion of synaptic vesicles[135,136]. In addition to this presynaptic role, NSF was also found to be a constituent of the PSD[137] and, intriguingly, it was shown to be enriched following transient ischaemia[138], which enhances synaptic transmission.

GluR2 was first shown to interact with NSF using the Y2H screen[139,140]. These findings were confirmed by the demonstration that GluR2 and NSF coimmunoprecipitate from the rat hippocampus along with α- and β-SNAPs in a complex analogous to the t-SNARE syntaxin-NSF-SNAP complex[141]. The binding site was identified as a ten–amino acid section of the GluR2 C-terminal domain[140,142] and a peptide of this binding site was found to be sufficient for the interaction to occur. Furthermore, this binding site on GluR2 was determined to show no similarity to other sites to which NSF binds, allowing functional experiments using this peptide. Partial inhibition of synaptic transmission was observed following perfusion of this peptide to postsynaptic sites, which indicates that NSF plays a role in a rapid-recycling pool of AMPARs but that there is also a pool of receptors that are not recycled through this mechanism. These experiments also allowed a calculation of a synaptic half-life of less than 10 min for these rapidly recycled receptors[140]. A further set of experiments using this peptide on cultured hippocampal neurones demonstrated both a decrease in the frequency but not the amplitude of AMPAR-mediated miniature excitatory postsynaptic currents and a significant reduction in AMPAR immunoreactive puncta on the surface[62], clearly demonstrating a role for NSF in the surface expression of AMPAR. An extension of this study focussing specifically on mechanisms of long-term depression found that its expression involves the removal from synapses of a pool of AMPAR controlled by the NSF–GluR2 interaction[143].

### *NARP*

NARP is a neuronal immediate early gene product, which is a member of a secretory family of proteins called the *pentraxins.* NARP was initially identified using a subtractive cloning strategy in a stimulated hippocampus since it remains elevated for nearly 24 h after a single episode of electroconvulsive seizure[144]. This extracellular protein appears to play a role in the synaptic clustering of AMPAR on some neurones[24]. NARP was also found on the surface at these synapses, and a significant amount of NARP was found in the media around the cells, in accordance with its membership in a family of secretory proteins. Transfection studies in spinal neurones demonstrated that synaptic NARP could be derived from both the pre- and postsynaptic neurone. This was confirmed by electron microscopy studies that revealed NARP in the presynaptic bouton associated with vesicles, in the cleft, and in postsynaptic structures. In addition, these transfected spinal neurones showed an increase in the number of excitatory synapses[24]. Repeated electroconvulsive seizures on the hippocampus caused NARP protein levels to remain elevated, about sixfold higher than basal levels, at 48 h after the last of a series of five or six electroconvulsive seizures given every other day. Therefore, sustained increases in NARP may contribute to changes in excitatory synaptic transmission induced by chronic neuronal stimulation[145].

### *Stargazin*

Stargazin is a mutated protein that is structurally related to the $\gamma$-1 calcium channel subunit present in the Stargazer mouse[146,147], an ataxic and epileptic mutant mouse that lacks functional AMPARs on cerebellar granule cells.

Stargazin interacts with both AMPAR subunits and synaptic PDZ proteins such as PSD-95. The interaction of stargazin with AMPAR subunits is essential for delivering functional receptors to the surface membrane of cerebellar granule cells, whereas its binding with PSD-95 and related PDZ proteins through a carboxy-terminal PDZ-binding domain is required for targeting the AMPAR to synapses[148]. Different stargazin isoforms are expressed in other neuronal cell types, including hippocampal pyramidal cells, leading to the hypothesis that they may perform a similar function in those cell types. In addition, expression of a mutant stargazin lacking the PDZ-binding domain in hippocampal pyramidal cells disrupts synaptic AMPARs, indicating that stargazin-like mechanisms for targeting AMPARs may be widespread in the central nervous system.

### *4.1N and 4.1G*

4.1 proteins are a family of multifunctional cytoskeletal components (4.1R, 4.1G, 4.1N, and 4.1B) derived from four related genes, of which 4.1N is expressed in neurones throughout the nervous system. 4.1N is enriched at synapses, and it colocalises with GluR1; see[149]. By analogy with the roles of 4.1R in red blood cells, these workers suggested that 4.1N may function to confer stability and plasticity to the neuronal membrane via interactions with multiple binding partners, including the spectrin-actin–based cytoskeleton, integral membrane channels and receptors, and MAGUKs[149].

A direct interaction between GluR1 and 4.1G and 4.1N proteins was subsequently demonstrated[150]. Both 4.1G and 4.1N bind to a membrane-proximal region of the GluR1 C-terminus and the site of interaction on 4.1G or 4.1N lies in a defined region in the C-terminal domain. Given this and its expression throughout the nervous system, 4.1N may serve to link GluR1 to the actin cytoskeleton as it also contains the binding site for actin complexes. This hypothesis is supported to some extent by the observation that disruption of actin filaments in cultured cortical neurones causes a down-regulation of GluR1 surface expression[150].

### *Lyn*

Lyn is a c-src–like, membrane-associated nonreceptor protein tyrosine kinase that is highly enriched in a subcellular fraction of nerve growth cones. In neurones, 1 to 2% of Lyn associates with AMPAR subunits through its SH2 and SH3 domains; see Hayashi et al.[151]. The precise site of interaction on AMPAR subunits was not established but is assumed to be intracellular. Protein tyrosine kinases link to the mitogen activated protein kinase (MAPK) signalling pathway and Lyn was shown by this study to link AMPAR activation, through this pathway, to activation of expression of brain-derived neurotrophic factor mRNA. Brain-derived neurotrophic factor has previously been shown to strengthen the efficiency of synaptic transmission[152,153], so this protein implicates AMPARs in a direct role in synaptic plasticity as well as in mediating rapid synaptic transmission.

### $G\alpha_{i1}$

AMPAR signalling (but not NMDAR signalling) in rat cortical neurones has been reported to involve a G-protein coupled to a protein kinase cascade; see Wang and Durkin[154]. Both NMDA and AMPA activated p42 MAPK in neurones, but only AMPA-induced MAPK was inhibited by pertussis toxin (an inhibitor of $G_i$ proteins). Furthermore, AMPA, but not NMDA, caused an association of a G-protein beta subunit with a Ras, Raf kinase, and MAP/ERK kinase–1 complex. These authors suggested that AMPAR activation could trigger MAPK activation via a novel mechanism in which G-protein beta gamma dimers released from Gα bind to a Ras protein complex, causing the activation of Ras, Raf kinase, MAP/ERK kinase–1, and finally MAPK[154]. However, no sites for G-protein binding have been identified on AMPARs. Nonetheless, these workers went on to propose that AMPAR activation can generate intracellular signals that are independent of $Ca^{2+}$ and $Na^{+}$ influx through ion channels. In the absence of intracellular $Ca^{2+}$ and $Na^{+}$, AMPAR stimulation inhibited pertussis toxin–mediated ADP-ribosylation of the Gα[155]. AMPA also inhibited forskolin-stimulated activity of adenylate cyclase in neurones, suggesting that $G_i$ proteins were activated. Moreover, coprecipitation experiments demonstrated that the modulation of the $G_i$ protein arose from the association of Gα (il) with the GluR1 subunit. These results suggest that, as well as acting as an ion channel, the AMPAR can exhibit metabotropic activity[155]. In retinal ganglion cells AMPAR activation has also been reported to modulate a G-protein that, in turn, suppresses the inward current through a cGMP-gated channel. This channel is activated by nitric oxide (NO). The AMPA-induced suppression of the cGMP-gated current was blocked by pertussis toxin, suggesting that AMPARs can exhibit a "metabotropic" activity that antagonises excitation evoked by NO[156].

## *KARs*

In a similar fashion to AMPAR, KARs exhibit a metabotropic function[157]. More specifically, the down regulation of the hippocampal inhibitory postsynaptic current by kainate is sensitive to pertussis toxin and independent of ion channel current. Furthermore, the action of kainate is prevented by calphostin C, a specific inhibitor of PKC, and reduced by inhibition of phospholipase C. The effect of kainate is occluded by phorbol esters and by increasing extracellular $Ca^{2+}$ but remains unaltered after inhibition or activation of PKA. These results are consistent with the activation of KARs stimulating a second messenger cascade involving inhibition of γ-aminobutyric acid release[157].

## CONCLUDING REMARKS

While significant advances have been made in identifying proteins that interact with AMPAR, and to a lesser extent KARs, the precise roles of these interactors and how they relate to each other remains unclear. Given that the abundance and distributions of interacting proteins vary between neuronal cell types, the mechanisms underlying glutamate receptor trafficking and surface expression are also likely to differ. A great deal more work needs to be done to define the developmental, activity-dependent, and steady-state processes that regulate changes in AMPAR- and KAR-mediated neurotransmission. Meeting this challenge represents a significant goal in Neuroscience that should lead the development of intervention and treatment strategies that may provide effective strategies for the treatment of excitotoxic and neurodegenerative diseases.
